# Culture-independent assessment of the indigenous microbial diversity of Raniganj coal bed methane block, Durgapur

**DOI:** 10.3389/fmicb.2023.1233605

**Published:** 2023-09-04

**Authors:** Mansi Chawla, Meeta Lavania, Nishi Sahu, Sudhanshu Shekhar, Nimmi Singh, Anand More, Magesh Iyer, Sanjay Kumar, Komal Singh, Banwari Lal

**Affiliations:** ^1^Environmental and Industrial Biotechnology Division, The Energy and Resources Institute, New Delhi, India; ^2^ONGC Energy Centre, New Delhi, India; ^3^Essar Oil and Gas Exploration and Production Limited, Durgapur, West Bengal, India

**Keywords:** coalbed methane (CBM), methanogenesis, microbial community, biogenic CBM enhancement, Raniganj coal block

## Abstract

It is widely acknowledged that conventional mining and extraction techniques have left many parts of the world with depleting coal reserves. A sustainable method for improving the recovery of natural gas from coalbeds involves enhancing the production of biogenic methane in coal mines. By taking a culture-independent approach, the diversity of the microbial community present in the formation water of an Indian reservoir was examined using 16S rRNA gene amplification in order to study the potential of microbial-enhanced coal bed methane (CBM) production from the deep thermogenic wells at a depth of 800–1200 m. Physicochemical characterization of formation water and coal samples was performed with the aim of understanding the *in situ* reservoir conditions that are most favorable for microbial CBM production. Microbial community analysis of formation water showed that bacteria were more abundant than archaea. Proteobacteria, Firmicutes, and Bacteroidetes were found as the most prevalent phyla in all the samples. These phyla play a crucial role in providing substrate for the process of methanogenesis by performing fermentative, hydrolytic, and syntrophic functions. Considerable variation in the abundance of microbial genera was observed amongst the selected CBM wells, potentially due to variable local geochemical conditions within the reservoir. The results of our study provide insights into the impact of geochemical factors on microbial distribution within the reservoir. Further, the study demonstrates lab-scale enhancement in methane production through nutrient amendment. It also focuses on understanding the microbial diversity of the Raniganj coalbed methane block using amplicon sequencing and further recognizing the potential of biogenic methane enhancement through microbial stimulation. The findings of the study will help as a reference for better strategization and implementation of on-site microbial stimulation for enhanced biogenic methane production in the future.

## 1. Introduction

Coal bed methane (CBM) is oftentimes regarded as the fuel that bridges the gap between conventional fossil fuels and sustainable energy sources. Methane is known to have a lesser adverse effect on the environment and human health due to its cleaner combustion and higher calorific value than coal. Both thermogenic and biogenic pathways can produce methane in coalbeds (Stolper et al., 2014). High temperatures and pressures (157–221°C) during coalification lead to the formation of thermogenic methane, whereas biogenic methane is predominantly produced by methanogens via microbial degradation of coal at moderate temperatures (50°C) (Wei et al., 2014).

A sizeable fraction of the world’s natural gas reserves is made up of coalbed methane (CBM), and it has been speculated that up to 20% of all natural gas, which includes CBM, emanates from microbes (Rice and Claypool, 1981). Enhanced CBM, also known as microbially enhanced CBM (MECoM), is the technique of stimulating microbes to produce more methane from currently producing wells. It may be possible to facilitate the microbial communities involved in the dynamic process of methanogenesis that produces CBM to further increase the methane production from the biodegradation of coal over timescales that are relevant to the industry. The lifespans of deficient microbial CBM wells could be increased if microbial CBM production could be enhanced, and possibly new microbial methane could be produced in sites with no history of gas production. In addition to the commercial gain, enhancing microbial methane production could also lower the adverse environmental effects of CBM production as the existing infrastructure would be used instead of drilling new wells when the old wells are depleted. Similar techniques might be utilized to produce methane in gas deficient shales from coal waste or deep or thin, potentially unmineable coal seams (Ritter et al., 2015).

It is the current belief that the abundance and diversity of the microbial community present in the coal seams are crucial to the production of biogenic methane in the coal beds. Research in the past has repeatedly shown that the interaction of several microbe groups involved in bio-methanation, such as fermentative bacteria, syntrophs, and methanogens, is necessary for the microbial mineralization of organic materials to methane (Strąpoć et al., 2011; Wawrik et al., 2012; Park and Liang, 2016; Wang et al., 2016). Numerous studies have thus far investigated the microbial community of coalbeds from various geographical regions, including Japan (Shimizu et al., 2007), the United States (Barnhart et al., 2013), Vietnam (Hoang et al., 2021), Canada (Lawson et al., 2015), and China (Guo et al., 2012). In contrast, not much is known about the microbial population in coalbeds located in geologically differing regions of India.

Therefore, the principal objective of this study is to fill the knowledge gap by providing a thorough analysis of the microbial community and their potential functions in the formation water collected from the Raniganj CBM block located in Durgapur, West Bengal. Further, to understand the role of geochemical factors in shaping the microbial diversity within the reservoir, the relationship between the microbial communities and geochemical factors is evaluated. The study also illustrated significant enhancement in the production of biogenic methane at the lab scale. To the best knowledge of the authors, this study is a prime investigation elucidating the diversity of microbial communities present in this region by 16S rRNA-based metagenomic analyses. Understanding the role of the indigenous microbial population in methanogenesis would help highlight the potential of comparable high temperature CBM reservoirs for biogenic methane production via *in situ* stimulation in the future.

## 2. Materials and methods

### 2.1. Study site and geographical setting

**Figure 1:**
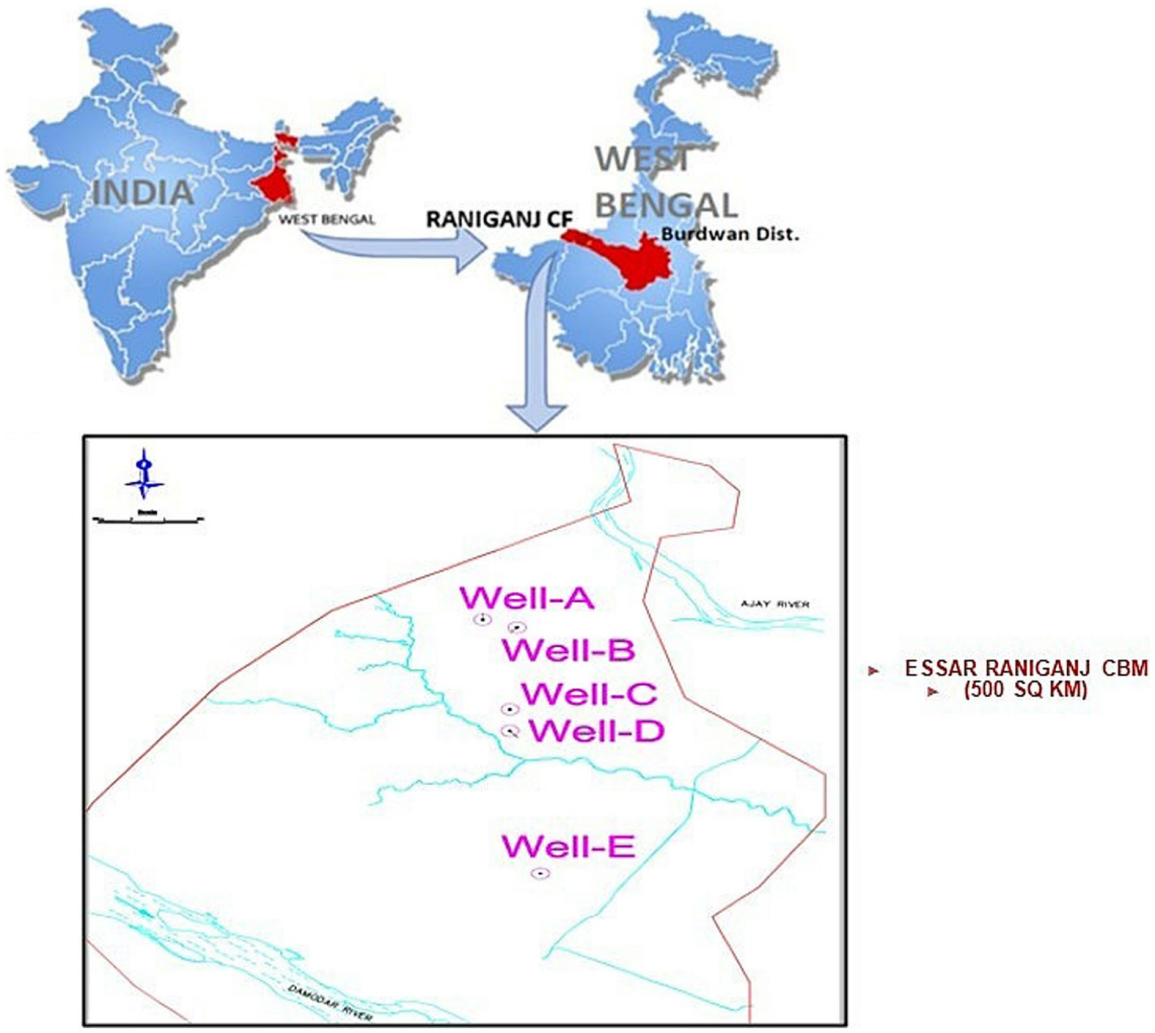
The Raniganj CBM (Coal Bed Methane) block located in the Burdawan district of West Bengal, India.

**Figure 2:**
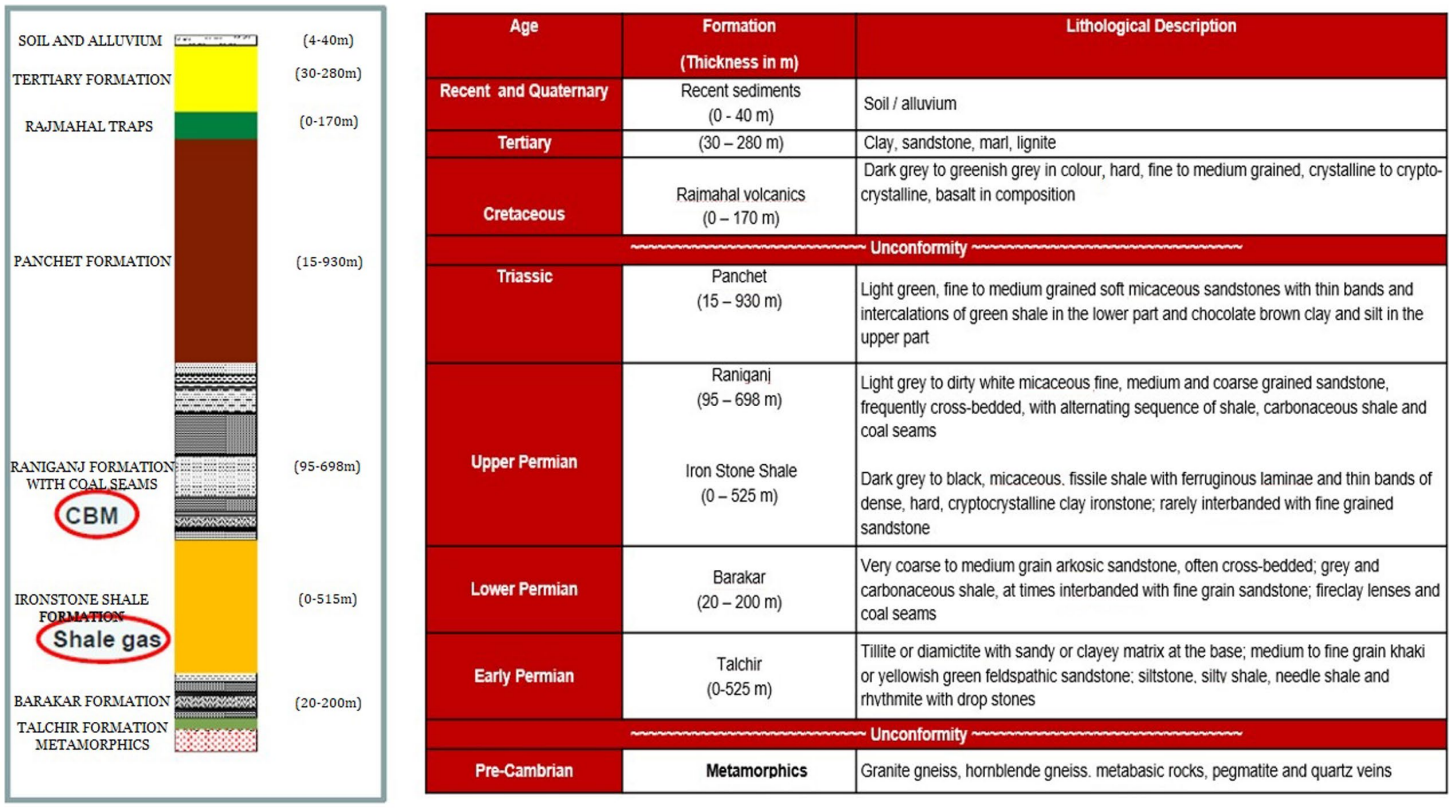
Generalized litho-stratigraphy of the basin.

Samples of formation water, gas, and coal were acquired from five CBM wells, namely Well-A, Well-B, Well-C, Well-D, and Well-E, present in ESSAR Oil’s Durgapur coal bed methane block, which is located in West Bengal, India. Figure 1 shows the coal block from which the coal wells were selected for this study. The Raniganj CBM (Coal Bed Methane) block is located in the Burdawan district of West Bengal, India. The block is spread over a 500 sq. km. area in the prolific Damodar Valley Basin with thick Permo-Carboniferous Gondwana coal seams. The majority of India’s coal reserves are found in the lower Gondwana stratigraphic sequence, which is of Permian age and encompasses the coal-rich Raniganj and Barakar measures. Figure 2 shows the generalized litho-stratigraphy of the basin, depicting that the effective thickness of the Raniganj formation with coal seams is at 95–698 m, which is seen to be related to high CBM productivity amongst all other assets of ESSAR Oil. The vast coalfields of Raniganj, Barakar, and Jharia hold enormous amounts of high-quality coal. On the basis of coal rank, coal maturity, available area, physicochemical properties of coal, CBM potential, depth of occurrence of coal, and geological age, the Indian coal basins are categorized into four kinds (Vedanti et al., 2020). The Raniganj coalfield is classified as Category I and has thick coal seams with high rank and maturity, as well as large reservoirs of methane gas and sorption capacity (Vishal et al., 2013). The field has been developed with over 340 production wells and an extensive network of infield pipelines; spread over 300 km, connecting the wells to four gas-gathering stations. Essar Oil & Gas Exploration and Production Limited (EOGEPL) is a significant natural gas supplier for the region and provides gas feed to many industries.

### 2.2. Site selection criteria

**Figure 3:**
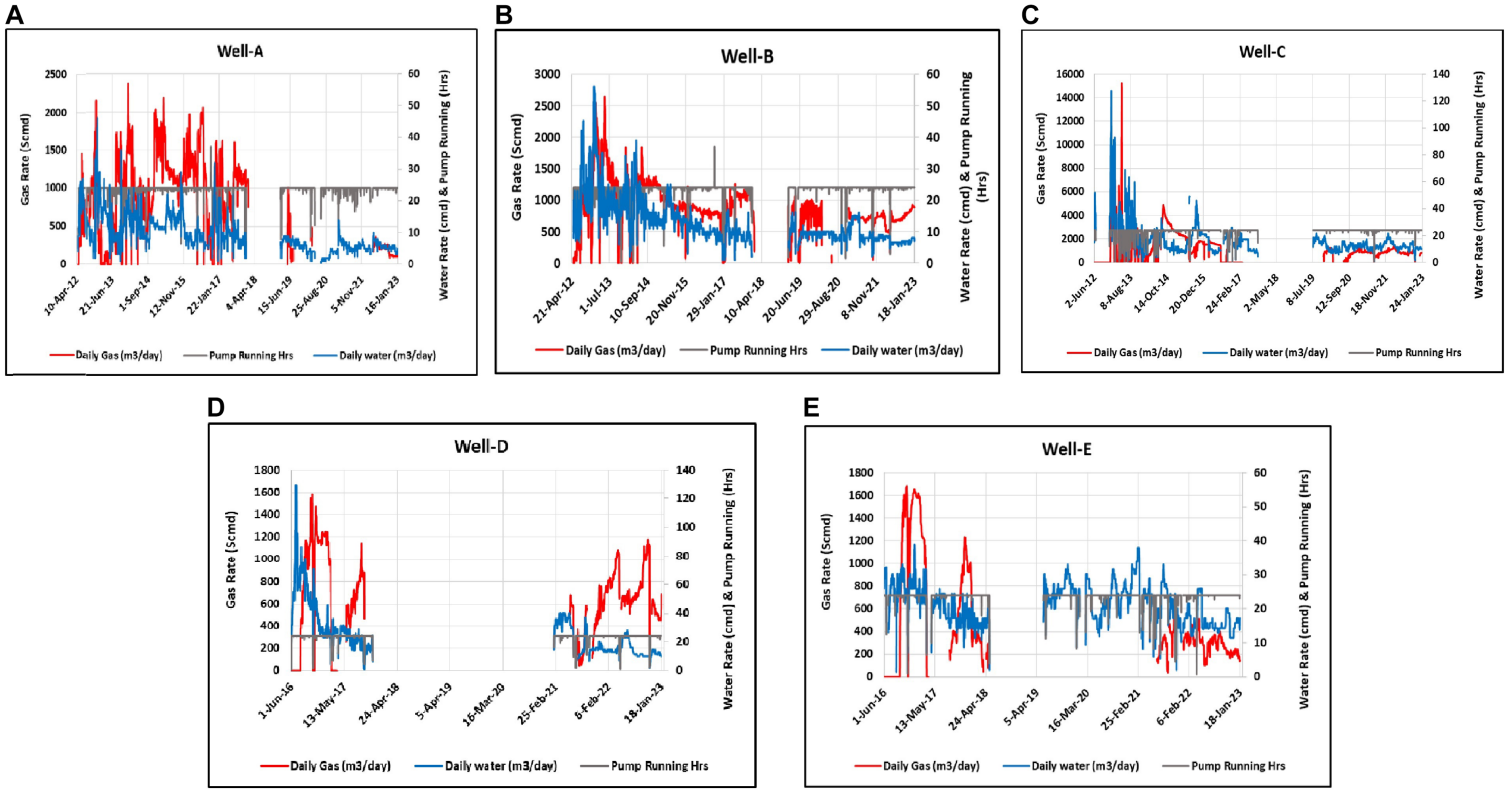
Daily production data of selected wells. **(A)** Complete production history of Well-A. **(B)** Complete production history of Well-B. **(C)** Complete production history of Well-C. **(D)** Complete production history of Well-D. **(E)** Complete production history of Well-E.

Understanding the *in situ* conditions and the history of the reservoir where the methane generation occurs is necessary, as these factors may affect the methane enhancement rates when the MECoM technology is applied in the reservoir. Producing wells with a gas and water production history were considered candidate CBM wells (Figure 3). The produced gas was studied for isotopic compositions to find evidence of biogenic, which could potentially be stimulated for enhanced methane production.

The majority of the coal-degrading microbial communities involved in methane production resides in the fractures (cleats) in coal seams and are typically 3–10 μm broad or at the interface of coal and surrounding rocks. Thus, further selection of the wells was made on the basis of feasibility tests performed on the field, which included testing the porosity and virgin permeability, as well as the post-fracture permeability and the half-fracture length. Essential criteria such as the bottom-hole temperature of the well and the pH and salinity of the formation water were also taken into account. The five selected wells had bottom-hole temperatures of less than 65°C, and the pH of formation water from all wells was less than 8, with salinity being less than 5%. The number of producing coal seams and the drilling depth of the wells were also considered important parameters in the selection process. The drilling depth of the selected wells is in the range of 901–1435 m, with the producing coal seams in the range of 5–8 having varying thicknesses between 0.3 and 5.82 TVD m.

### 2.3. Sampling

The collection of samples was conducted using a polyvinyl chloride pipe that was 1 m long with a diameter of 10 cm, sealed at the bottom, and closed with an open steel mesh at the top. At the field site, a bleach solution was used to sterilize the pipe and mesh enclosure prior to lowering them to the bottom of the well on a wireline. Sterilized 1000 mL anaerobic serum bottles sparged with nitrogen were used to collect the sample upto the brim and then sealed with airtight rubber septa and aluminum crimps. Samples were also collected for on-site testing of pH and conductivity. The sample bottles were then brought to the laboratory for further analysis. Conforming to the method outlined by Rathi et al. (2015), formation water samples were also collected from the well-heads of the selected candidate wells, based on selection studies, and transferred into sterile 100 mL anaerobic serum bottles containing 1 mL of 2% Na_2_S. All the samples were brought to the laboratory in under 48 h, kept at 4°C, and then promptly processed for microbial analysis. Additionally, produced gas samples collected from the well heads were processed for compositional and isotopic analysis. A representative coal sample was drilled from the CBM block of Essar Oil, located in Durgapur, West Bengal, and was used as representative coal for all CBM wells involved in the study.

### 2.4. Physicochemical analysis

#### 2.4.1. Formation water

Each well’s collected formation water samples underwent physicochemical analysis for pH and Electrical conductivity at room temperature per the American Petroleum Institute (API) standards. The presence of heavy metals such as arsenic, cadmium, chromium, copper, and Fluoride was also estimated as per the standard methods (IS3025 PT 37:1988, APHA3100(B), APHA3500(B), APHA3111(B), APHA4500 (F-D)) respectively. The presence of cation [calcium APHA 3500 (B)] and anion [chloride: APHA 4500, nitrate: IS 3025, phosphate: APHA 4500 (D), and sulfate APHA 4500 (E)] was also estimated in the formation water.

#### 2.4.2. Coal

The detailed analysis of coal in terms of ash, moisture, volatile matter, and fixed carbon, along with the specific carbon, hydrogen, nitrogen, sulfur, and oxygen (CHNSO) profile, was determined as per the guideline of ASTM standards.

#### 2.4.3. Carbon stable isotopes of collected gas

A critical tool for understanding the origin of CBM gas is the carbon isotope composition study. The molecular and isotopic compositions of the gases collected from the Raniganj field were studied by the Keshava Deva Malaviya Insititute of Petroleum Exploration, Oil and Natural Gas Corporation Ltd., according to the method stated by Wang et al. (2019).

### 2.5. Genomic analysis of formation water from the Raniganj block

Formation water samples were processed for genomic DNA extraction in order to evaluate the capacity of the thermogenic CBM reservoir to produce biogenic methane. 500 mL of each formation water sample was run through a filter membrane (0.22 μm) to extract genomic DNA. Coal particles were suspended in the formation water. All the microbes adhering to the coal particles that had remained on the filter were processed using the DNeasy Power Water kit for DNA extraction. The protocol provided by the manufacturer was followed for DNA extraction; quantification and analysis of DNA were performed using a NanoDrop spectrophotometer. The extracted DNA samples that exhibited high quality with A260/280 ratio between 1.8 and 2.0 and concentrations greater than 50 ng/μl were taken up further for sequencing.

To understand the overall diversity of the microbial population, extracted DNA of all five well samples was processed for PCR amplification using universal bacterial primers (27F and 1492R) as described by Rathi et al. (2019), V3-V4 amplicon sequencing was performed on the Illumina MiSeq 2500 platform by Medgenome Pvt. Ltd. The 16S rRNA gene sequences were checked for purity with the Check-Chimera program, and OTU picking at 97% sequence similarity thresholds (Open reference method) were performed. Data rarefaction, cumulative sum scaling (CSS), low variance, and low count filtering were carried out before downstream analysis. These pre-processing steps enabled to avoid the sequencing depth bias for better comparative analysis (Rajeev et al., 2021).

The microbial analysis was done in the software MicrobiomeAnalyst (Dhariwal et al., 2017). The prediction of the functional profiles of bacterial taxa based on 16S rRNA sequencing data was performed with the Metagenassist software using the amplicon sequencing data according to the Silva-132 database (Arndt et al., 2012). This tool was used to predict functional community profiles based on 16S rRNA data to outline microbial metabolism in the studied samples in the Raniganj coal bed formation water. Canonical correspondence analysis (CCA) and correlation analysis were used to analyze the relationship between the geochemical factors and taxonomic groups at the class level using software PAST 4.03 (Arndt et al., 2012).

The metagenome sequence reads of the samples from the candidate wells, Well-A, Well-B, Well-C, Well-D, and Well-E, were submitted to the NCBI archive under the BioProject accession number PRJNA965902.

### 2.6. Enrichment of a methane-producing microbial consortium from the formation water

Methane-producing microbes were selectively screened from the collected formation water samples (10% (v/v)) in the laboratory by enriching them in methanogen-specific nutrient media containing 1% (w/v) coal as the carbon source (Rathi et al., 2015). The composition (per liter) included K_2_HPO_4_; 0.4 g, KH_2_PO_4_; 0.2 g, MgCl_2_.6H_2_O; 0.1 g, NaCl; 0.5 g, NH_4_Cl; 0.2 g, NaHCO_3_; 0.2 g, and Yeast extract; 0.5 g. The pH was then adjusted to 7.0 ± 0.2. 100 μL of Resazurin was added to the media as an oxygen indicator. The media was boiled for 10 min and allowed to cool down while being purged with nitrogen to remove the dissolved oxygen. The remaining dissolved oxygen was then subsequently removed by adding 0.5 g L-cysteine hydrochloride, making the environment of the media completely oxygen-free. A volume of 30 mL media was dispensed into 60 mL anaerobic serum bottles and flushed again with N_2_. The bottles were sealed with butyl rubber stoppers and crimped with aluminum caps. The sealed and pressurized bottles were autoclaved before inoculation for 15 min at 121°C. The bottles that were not inoculated were considered as control.

Methane generation in incubated culture bottles was monitored during the enrichment process by collecting 0.5 mL of headspace gas samples from the serum bottles via a gas-tight syringe and quantifying the anticipated headspace gases, such as methane, carbon dioxide, nitrogen, and hydrogen, using gas chromatography. All experiments were run in a triplicate set. The data points are the average of triplicate±standard deviation (<5% of average).

## 3. Results

### 3.1. Understanding the role of limiting factors for biological methane production via physio-chemical analysis

Physio-chemical analysis of formation water collected for this study is shown in Table 1. The pH was seen to be in the range of 7.0–8.3, suggesting suitable conditions in the reservoir for biogenic methane production. It is notable that similar recent studies have reported the formation water samples in their studies to be pH range up to 8.3 (Hoang et al., 2021). The concentration of heavy metals, including Arsenic (As^5+^), Cadmium (Cd^2+^), Chromium (Cr^2+^), Copper (Cu^2+^), Zinc (Zn^2+^), Silver (Ag^2+^), and Nickel (Ni^2+^), was found to be below detectable range, signifying a non-toxic environment for the indigenous microbial community to flourish and produce methane. The presence of Sulfate (SO_4_^2−^), Fluoride (F^−^), and Iron (Fe^2+^) was found and is shown in Table 1. In this study, the concentration of sulfate ions in the formation water also corroborates what was seen in past investigations in Indian reservoirs (Rathi et al., 2019).

**Table 1:** Physicochemical characterization of the formation water samples.

Heavy metals (mg/l)	Test methods	Well – A	Well – B	Well – C	Well – D	Well – E
Iron (Fe^2+^)	APHA3100(B)	0.31 mg/L	0.3 mg/L	0.9 mg/L	9.2 mg/L	2.3 mg/L
Fluoride (F^−^)	APHA4500 (F-D)	0.61 mg/L	1.7 mg/L	2.1 mg/L	0.89 mg/L	3.8 mg/L
Sulfate (SO_4_^2−^)	IS3025 pt 24:1986	3.93 mg/L	7.2 mg/L	6.0 mg/L	4.78 mg/L	6.4 mg/L
pH		8.3 at 25°C	7.90 at 25°C	7.80 at 25°C	8.3 at 25°C	7.0 at 25°C
Electrical conductivity		5.3 mS/cm	4.9 mS/cm	2.9 mS/cm	12.48 mS/cm	3.3 msS/cm
Total dissolved solids		2717 mg/L	2200 mg/L	3200 mg/L	6196 mg/L	3900 mg/L

The proximate analysis of coal of the seams from all selected CBM wells was carried out (Table 2). The coals are of low moisture (1.71–4.69%) and medium ash (11.38–19.25%) with an average of 33% volatile matter and approximately 50% fixed carbon. These results indicate that the ASTM rank of the coal is high volatile ‘A’ bituminous (HVAB). A laboratory feasibility study was conducted by Fallgren et al. (2013), focusing on the comparison of coal ranks for biogenic methane production via the enrichment of microbial consortia. The study indicated that the rate of methane production was higher in the case of bituminous coal as compared to lower-rank coals. Ultimate analysis results indicated low sulfur content (0.32–0.47%) with up to 67% carbon and up to 4.14% hydrogen.

**Table 2:** Proximate and ultimate analysis of coal samples from Durgapur fields.

Proximate analysis	Standards	Well – A	Well – B	Well – C	Well – D	Well – E
Inherent moisture (%)	IS:1350 (Pt-I) 1984 Reaff-2007	0.13	0.01	0.11	0.13	0.17
Ash (%)	IS:1350 (Pt-I) 1984 Reaff-2007	19.25	12.51	11.38	12.51	13.59
Total moisture content (%)	IS:1350 (Pt-I) 1984 Reaff-2007	3.49	4.69	2.50	3.73	1.71
Volatile matter (%)	IS:1350 (Pt-I) 1984 Reaff-2007	33.25	31.91	32.64	33.97	32.14
Fixed carbon (%)	IS:1350 (Pt-I) 1984 Reaff-2007	44.14	51.26	53.59	49.92	52.73
Ultimate analysis						
Carbon (%)	IS:1350 (Pt-IV/Sec-I) 2011	57.19	62.33	65.97	64.77	65.70
Hydrogen (%)	IS:1350 (Pt-IV/Sec-I) 2011	3.87	4.03	4.14	4.08	4.06
Nitrogen (%)	IS:1350 (Pt-IV/Sec-I) 2011	1.40	1.50	1.26	1.28	1.25
Sulfur (%)	IS:1350 (Pt-III) 2011	0.32	0.37	0.43	0.47	0.33
Oxygen (%)	IS:1350 (Pt-II) 1970 Reaff-2002	12.68	13.72	13.29	12.04	12.17
Mineral matter (1.1XA) (%)	IS:1350 (Pt-I) 1984 Reaff-2007	21.18	13.37	12.52	13.76	14.95
Moisture content (%)	IS:1350 (Pt-I) 1984 Reaff-2007	3.36	4.68	2.39	3.60	1.54
Gross calorific value, Kcal/Kg	By difference	5376	5859	6202	6088	6108

### 3.2. Molecular and isotopic compositions of studied gases

Analyzing the carbon isotopic fingerprints (*δ*^13^ C-CH_4_) produced by biogenic and thermogenic CH_4_ allows one to determine the source of the gas (Schoell, 1980). Biogenic CH_4_ can have *δ*^13^ C values between-110% and-40%, but the values of thermogenic CH_4_ carbon stable isotopes are between −50% and −20% (Whiticar, 1999). The molecular and isotopic compositions of the studied gases from the Raniganj field indicate their mixed origin. The gases are also dry in nature (C1/(C2 + C3) >50) (Bernard et al., 1976). The significant difference in stable carbon isotope ratios of methane and ethane (~20%), and the presence of isotopically heavier carbon dioxide (6.6–15.5%) indicate its association with biogenic processes (Kinnon et al., 2010), particularly H2 utilizing hydrogenotrophic methanogens. The results of the molecular and isotopic compositions are provided in detail in Table 3.

**Table 3:** Molecular and isotopic compositions of studied gases.

Well	Chemical composition (% Mol)											Stable Carbon isotopic values (*δ*^13^C%)			
	C_1_	C_2_	C_3_	iC_4_	nC_4_	iC_5_	nC_5_	C_6_	C_2_+	CO_2_	N_2_	*δ*^13^C_1_	*δ*^13^C_2_	*δ*^13^C_3_	*δ*^13^CO_2_
A	98.99	0.08	0	0	0	0	0	0	0.08	0.30	0.6	−49.6	–	–	–
B	98.88	0.13	0.12	0.03	0.08	0.03	0.05	0.04	0.47	0.42	O.2	−50.3	–	–	8.2
C	98.27	0.99	0.08	0.01	0	0	0	0	1.09	0.42	0.2	−43.0	−25.7	−26.6	15.5
D	98.33	0.23	0.05	0.01	0	0	0	0.01	0.30	0.49	0.9	−42.0	−20.2	–	15.4
E	98.67	0.2	0.02	0	0	0	0	0	0.22	0.21	0.9	−49.7	−26.0	–	6.6

### 3.3. 16S rRNA amplicon sequencing depicting the relative abundance of microbial diversity

In this investigation, five coal bed methane wells were chosen for sampling as they are representative examples of relatively high temperature coal bed methane reservoirs with a bottom-hole temperature of about 50°C. The 16S rRNA gene clone library was created from the formation water samples of the CBM wells in order to study the microbial population of these high temperature thermogenic CBM wells and their relationship in connection to the potential for biogenic methane generation. The samples had a total of 153,403 effective read counts of the 16S rRNA gene amplicons. The rarefaction curve plotted between species richness and sequence sample size has been shown in Supplementary Figure 1.

**Figure 4:**
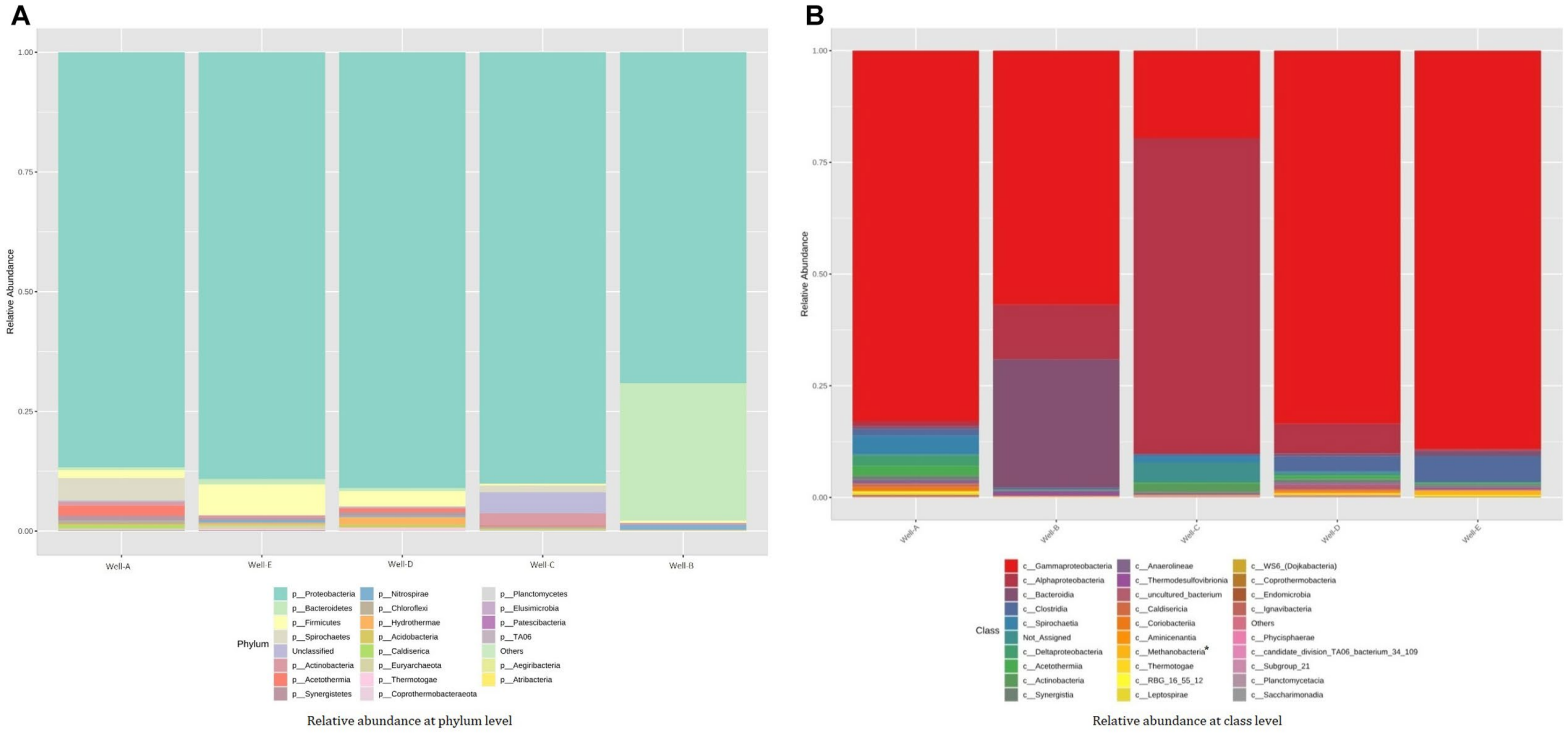
Stacked bar plot representing the relative abundance assessed by 16S rRNA-gene amplicon sequencing at **(A)** phylum-level and **(B)** class level (* denotes archaeal group).

The amplicon sequencing reveals the relative abundance of the microbes present in the studied wells, showing both archaeal and bacterial diversity (Figure 4). Proteobacteria, Firmicutes, Bacteroidetes, and Actinobacteria were the most prevalent phyla in all the samples. The formation water from the thermogenic high temperature CBM reservoir in this study demonstrated that the abundance of Proteobacteria was primarily composed of Gammaproteobacteria and Alphaproteobacteria. Other than the ubiquitously dominant phyla in the reservoir, Well-A shows a relatively high abundance consisting of Spirochaetes, Acetothermia, Synergistetes, Chloroflexi, and Caldiserica. Well-B shows a relatively high abundance of Nitrospirae and Chloroflexi. A significant proportion of uncultured bacteria is found in the Well-C, followed by Spirochaetes, Acetothermia, and Chloroflexi, among other relatively less dominant phyla. Well-D is also found to be abundant with Acetothermia, Spirochaetes, Caldiserica, Chloroflexi, and Synergistetes, along with Hydrothermae, Coprothermobacteraeota, Acidobacteria, and Thermotoga. In the case of Well-E, the presence of Euryarchaeota, Nitrospirae, and Synergistetes is seen among other dominant phyla.

The taxonomic analysis at the class level shows the relative abundance of class *Gammaproteobacteria*, *Alphaproteobacteria*, and *Deltaproteobacteria*. The relative abundance pattern also showed the presence of class *Bacteroidia* and *Clostridia* in all the samples. Class *Methanobacteria* was the methanogenic group present in the samples.

**Figure 5:**
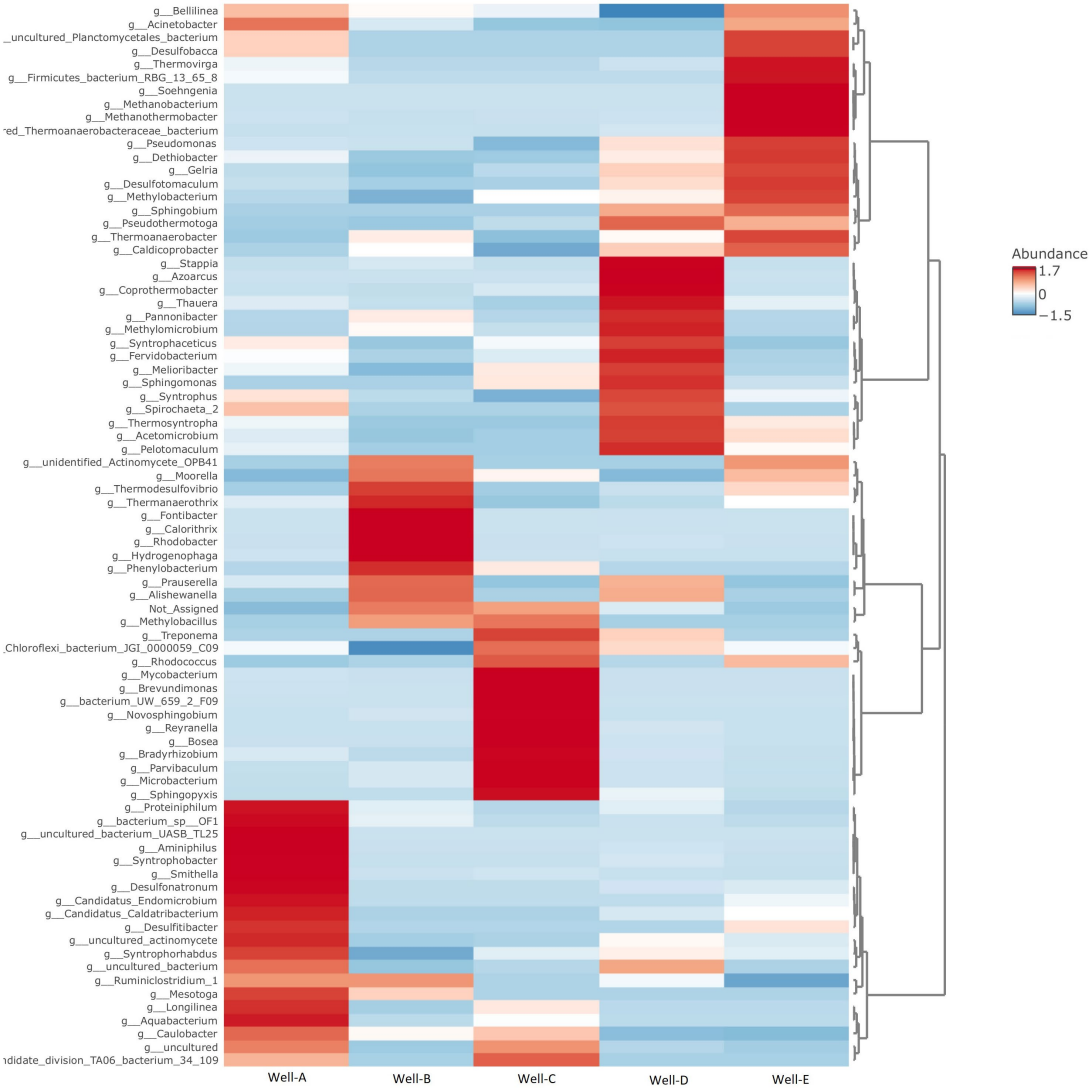
Heat map representing the relative abundance of microbial communities at the genus level, as seen in the formation water samples of selected CBM wells (Darker colors indicate a greater percentage of abundance than lighter colors).

At the genus level (as shown in Figure 5), the microbial community found in well number Well-A has a relative abundance of *Smithella, Aquabacterium, Desulfonatronum, Syntrophobacter, Caulobacter, Proteiniphium*, and *Desulfitibacter*, among others. The relative abundance, as seen in Well-B, majorly includes *Thermodesulfovibrio, Thermanaerothrix, Hydrogenophaga, Phenylobacterium*, and *Caulobacter*. Well-C also shows a dominant abundance of *Caulobacter*, along with *Methylobacillus, Brevundimonas, Novosphingobium, Parvibaculum, Bosea, Microbacterium, Sphingopyxis, Treponema, Bradyrhizobium, Rhodococcus, and Mahella*. In the case of Well-D, the most dominant community consists of *Methylomicrobium, Sphingomonas, Coprothermobacter, Thauera, Spirochaeta, Azoacrus, Pannonibacter, Stappia*, and *Acetomicrobium*. The microbial community found in well number Well-E majorly consists of *Methanobacterium, Methanothermobacter, Pseudomonas, Thermoanaerobacter, Acinetobacter, Methylobacterium, Thermovirga, Desulfobacca*, and *Desulfotomaculum*. *Methanobacterium* and *Methanothermobacter*, two well-known hydrogenotrophic methanogens, were the most prevalent archaeal groups found in Well-E.

### 3.4. Predictive functional analysis

**Figure 6:**
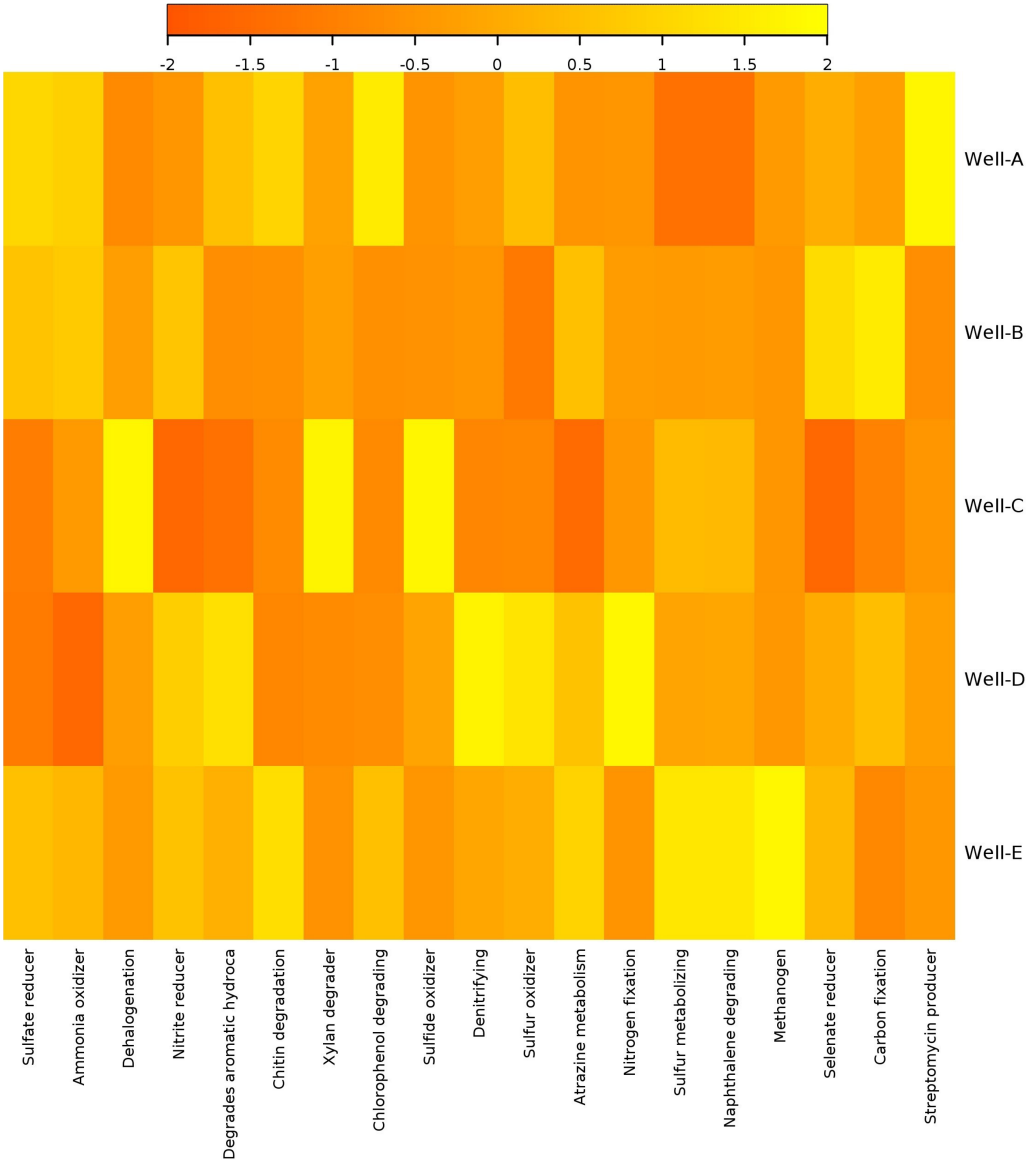
Heatmap showing relative function abundance predictions of the microbial communities based on metagenassist analysis.

The heatmap of predictive functional analysis shows the presence of methanogenic functions in all the wells suggesting the potential for enhancement in CBM (Figure 6). Among all the samples, the highest methanogenic activity is present in Well E, which is also supported by microbial diversity analysis. There is a high abundance of aromatic degradation in wells D, E, and A. The analysis also showed the existence of sulfate-reducing metabolism in the CBM wells. The abundance of the sulfate reducers was found to be lower than the methanogens in wells A, B, and C. It also reveals the presence of chitin and xylan degraders.

### 3.5. Understanding the effects of physicochemical parameters of formation water on microbial community

**Figure 7:**
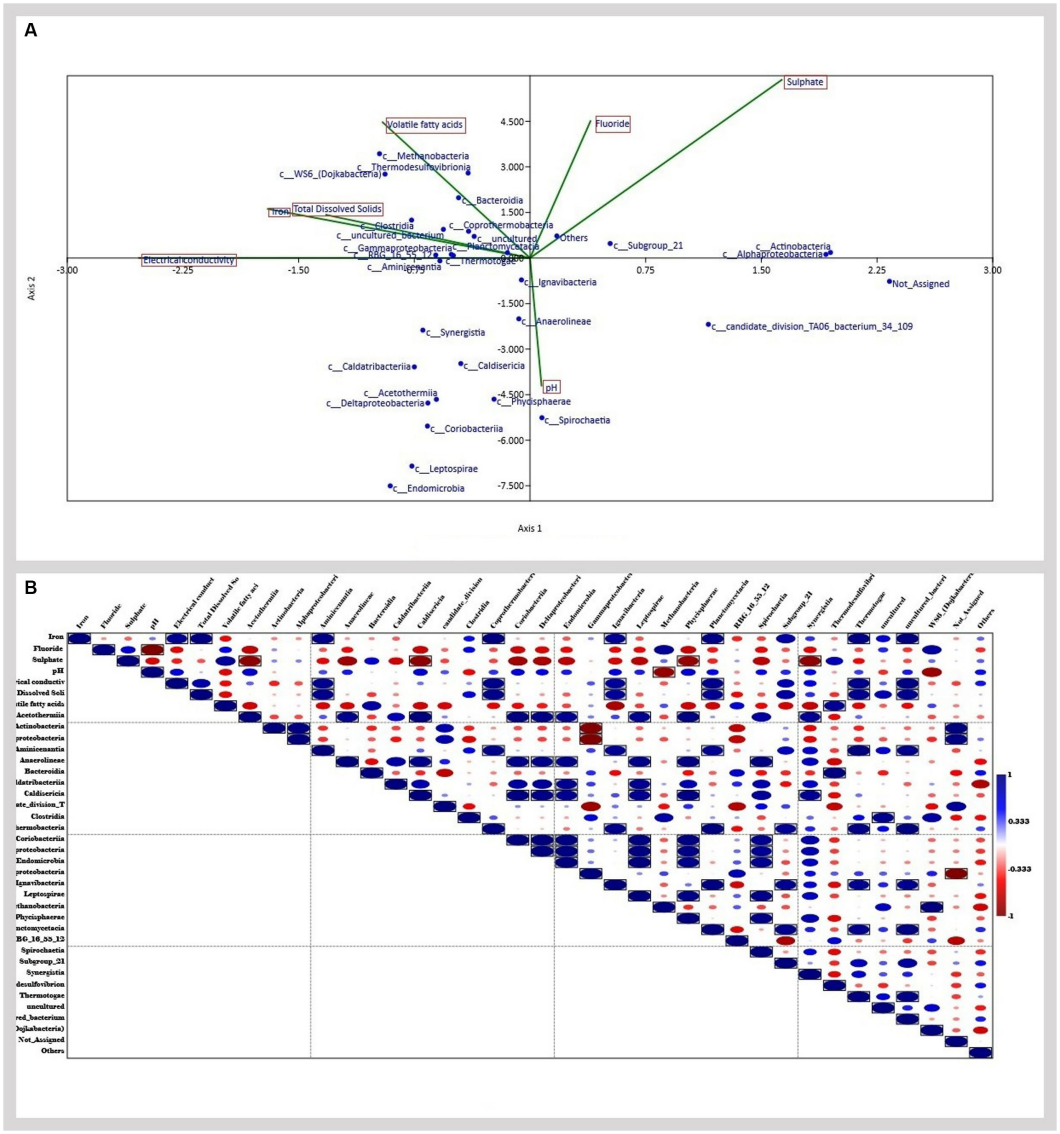
**(A)** Canonical correspondence analysis (CCA) analyzing the relationship between geochemical factors and microbial diversity **(B)** correlation analysis.

In the present study, Canonical correspondence analysis (CCA) was used to analyze whether correlation in microbial diversity is related to geochemical factors. According to the CCA analysis (Figure 7A), class *Clostridia, Gammaproteobacteria, Aminicenantia, Coprothermobacteria, Planctomycetacia, Methanobacteria*, and *Thermodesulfovibriona* were found to be related. This is further verified by the correlation analysis (Figure 7B), which showed a significant correlation between Iron, Electrical conductivity, and TDS with classes *Aminicenantia, Coprothermobacteria, Planctomycetacia, Thermodesulfovibriona*, and *Ignavibacteria*. The results show how geochemical factors impact the diversity and distribution of microbes in the environment.

### 3.6. Quantifying the methane gas generated in the laboratory by the consortium via coal bioconversion

**Figure 8:**
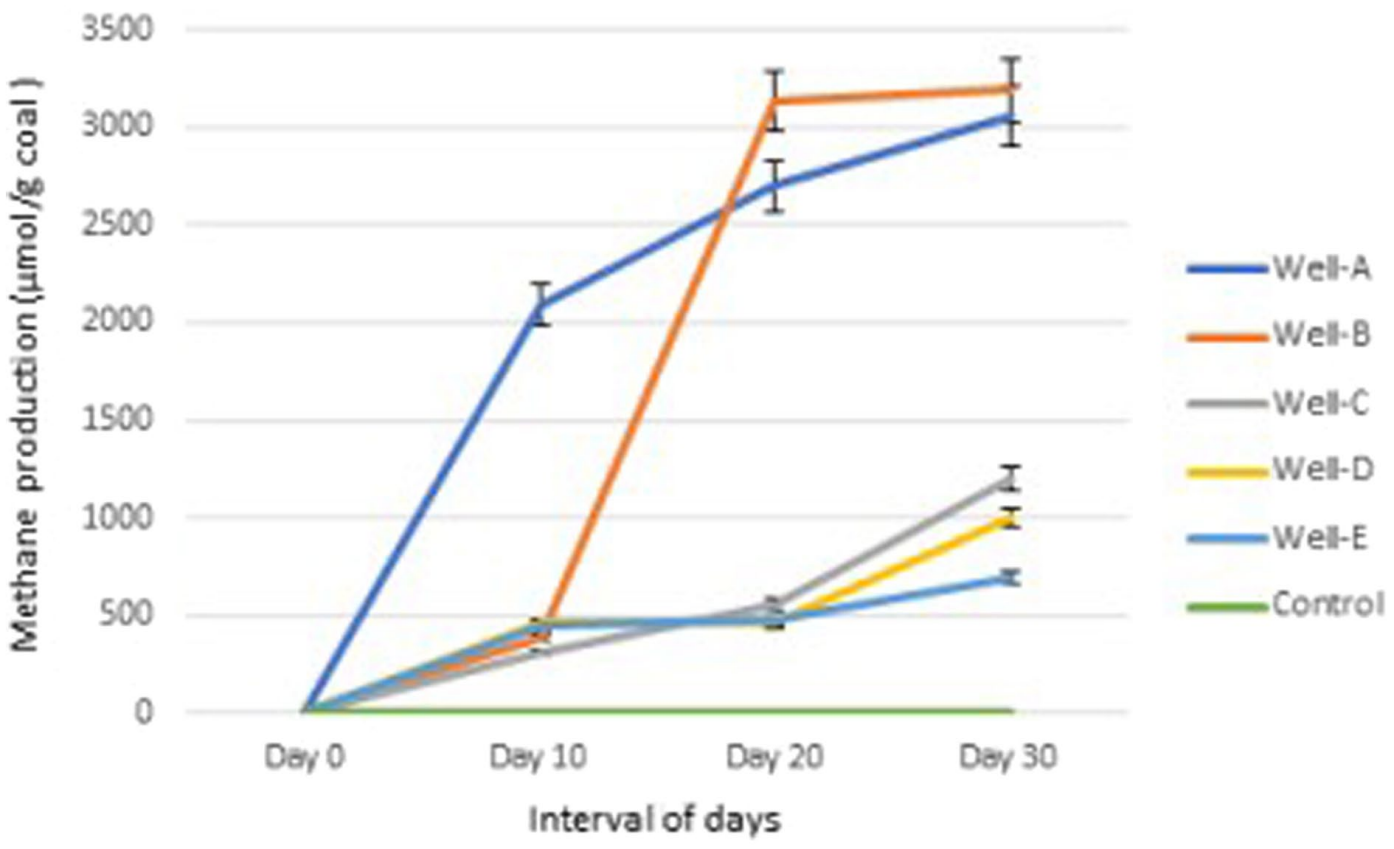
Methane production trend in the laboratory by the methanogen-specific consortium.

The amount of methane gas generated in the headspace of the experimental bottles containing the modified MPB nutrient media was quantified using gas chromatography. 3063.35 μmol CH_4_/g coal, 3190.64 μmol CH_4_/g coal, 1202.15 μmol CH_4_/g coal, 1004.28 μmol CH_4_/g coal, and 697.09 μmol CH_4_/g coal was observed to be produced by the thermophilic methanogenic consortium inoculated with formation water from well-A, well-B, well-C, well-D, and well-E, respectively, after 30 days of incubation (Figure 8). The experimental control media bottles containing 0.3 g coal under similar conditions showed no methane production at 55°C after 30 days.

## 4. Discussion

Numerous studies have examined the microbiological communities of coal/formation water for comprehending the methanogenic potential of coal beds in the USA, Canada, Japan, and Australia (Mochimaru et al., 2007; Shimizu et al., 2007; Green et al., 2008; Klein et al., 2008; Li et al., 2008; Strąpoć et al., 2008; Midgley et al., 2010; Penner et al., 2010). These microbial communities detected in coal beds have undergone culture-independent investigations that have revealed the identities of many bacterial species that are key players in the biodegradation of coal as well as archaea that produce methane via hydrogenotrophic or acetoclastic pathways. In this investigation, the 16S rRNA Amplicon sequencing revealed the relative abundance of the microbes present in the wells, showing that the most abundant phyla included Proteobacteria, Firmicutes, Bacteriodetes, and Actinobacteria. These phyla have been reported to be involved in the biotransformation of coal into methane in earlier studies (Strąpoć et al., 2011; Singh et al., 2012). The presence of Gammaproteobacteria and Alphaproteobacteria in the wells indicated different hydrocarbon-degrading pathways. It is widely recognized that bacteria from the phylum Alphaproteobacteria can break down hydrophobic compounds such as polycyclic aromatic hydrocarbons (Meslé et al., 2013). According to certain reports, Gammaproteobacteria can break down hydrocarbons when nitrate serves as the electron acceptor (Alain et al., 2012). In the past, Bacteroidetes have also been mentioned as pentachlorophenol degraders (Singh et al., 2012). The relative abundance shows the dominance of Euryarchaeota in Well-E. Euryarchaeota majorly consists of acetoclastic and hydrogenotrophic methanogens. Evidence suggesting that syntrophic acetate oxidation is coupled with hydrogenotrophic methanogenesis in the high temperature petroleum reservoirs, such as the Yabase oil field (Japan), has also been documented (Mayumi et al., 2011). Similar to the present study, a dominance of Spirochaetes has also been found by Singh et al. in the Jharia coal reservoir and in the coal bed of the Illinois basin (Strąpoć et al., 2008). As seen in the study by Green et al. (2008), the methanogenic consortium in the Powder River Basin included Spirochaetes in addition to Firmicutes, which predominated the bacterial community in coal bed methane wells there.

The relative abundance of class *Gammaproteobacteria, Alphaproteobacteria*, and *Deltaproteobacteria* in the taxonomic analysis of our samples at the class level is of significance, as members of *Gammaproteobacteria* and *Alphaproteobacteria* have been studied to be involved in the degradation of various organic compounds, including petroleum hydrocarbons (Zhou et al., 2020; Sudiana et al., 2022). Members of *Deltaproteobacteria* can anaerobically metabolize a wide variety of normal, iso-, and cyclic alkanes, as well as mono- and polycyclic aromatic hydrocarbons (Davidova et al., 2018). The hydrocarbonoclastic *Deltaproteobacteria* can take part in syntrophic consortia that ultimately transmit reducing equivalents to methanogenic Archaea without a respiratory electron acceptor. This shows their role in methane generation (Davidova et al., 2018). The presence of class *Bacteroidia* and *Clostridia* in all the samples is noteworthy, as previous studies have shown significant enhancement in methane production in consortia rich in *Bacteroides* and *Clostridium* (Tukanghan et al., 2021). Also, class *Clostridium* contains many hydrogen-producing bacteria, and since hydrogen is the limiting factor in the hydrogenotrophic mode of methanation, their presence is highly significant for enhanced CBM production (Palacios et al., 2021; Wang and Yin, 2021). The presence of Methanobacteria in the samples suggests hydrogenotrophic methanogenesis as a significant pathway of methane generation in our study.

There is a noteworthy variation in the genus level abundance within the reservoir. We speculate that this variation is due to the age of the coalbed, the depth of sampling, geological conditions, and the availability of substrates in the coalbed and formation water (Barnhart et al., 2016). Well-A is rich in *Smithella* and *Syntrophobacter*, which are syntrophic bacteria. These bacteria have been associated with coal degradation and with providing a substrate for methanogens to produce methane from mesophilic coal reservoirs in earlier research (Strąpoć et al., 2011; Singh et al., 2012). Well-B shows the presence of *Hydrogenophaga*, a typical hydrogen-utilizing bacteria which is also able to utilize biphenyl. In the study by Wei et al. (2014), the most dominant genus identified in abandoned coal mines was *Hydrogenophaga*. Another study highlighted that *Hydrogenophaga*, which was found in an oil-degrading consortium, is capable of metabolizing 4-aminobenzenesulfonate (Beckmann et al., 2011). It has been found in coal samples from the Liulin District (Guo et al., 2012) and in methanogenic enrichment modified with coal (Beckmann et al., 2011). The presence of *Phenylobacterium* was also abundant, and it also has been detected in the biogenic coal bed methane reserves of the Southern Qinshui Basin, China (Guo et al., 2014). The presence of sulfate-reducing bacteria such as *Thermodesulfovibrio, Desulfotomaculum*, and *Desulfitibacter*, which are known to be capable of degrading volatile organic matter (i.e., hydrocarbons and organic acids) in the presence of sulfate as the terminal electron acceptor, is attributed to the presence of sulfate ions and their concentration in the formation water. The prevalence of *Methylobacillus*, a methanotrophic bacterium, in the formation water of Well-C correlates with the findings of the study by Zhang et al. (2018) that methanogenic and methanotrophic pathways are present in the formation water of the coal reservoir, responsible for the carbon metabolism.

*Thauera* is amongst the most abundant in Well-D and is known to be a nitrate-reducing, hydrocarbon-degrading bacterium. It has been found in several other basins, including the Jharia basin (Singh et al., 2012), the Surat basin in Australia (Li et al., 2008), as well as the coal beds of Western Canada (Penner et al., 2010). The presence of *Coprothermobacter* is also notable in this study. *Coprothermobacter* sp. of the *Thermodesulfobiaceae* was previously identified as a thermophilic, chemo-organotrophic proteolytic acetogen in methanogenic enrichment culture from a thermophilic digester (55°C) (Ollivier et al., 1985). These proteolytic bacteria can convert yeast extract protein into acetate, hydroxyl, and carbon dioxide, as well as other acids in lower amounts, like isobutyric, isovaleric, and propionic acids. A recent study by Kobayashi et al. (2012) also claimed that *Coprothermobacter* works as a possible syntrophic acetate oxidizer in high-temperature methanogenic enrichment cultures. Moreover, Singh et al. noted that *Coprothermobacter* was dominant in their methanogenic enrichment culture.

The prevalence of *Methanobacterium* and *Methanothermobacter* in Well-E indicates that hydrogenotrophic pathways constitute the primary means of methane production. The presence of *Methanothermobacter*, which is also a thermophilic CO_2_-reducing methanogen, was found in similar studies, including the coal reservoir of Ruhr Basin, Germany, a biogenic methane-producing source, and the Powder River Basin, USA (Flores et al., 2008). The predominance of the genera *Pseudomonas* has been reported for hydrocarbon degradation and has been found in the coal seams of Western Canada (Penner et al., 2010). Similarly, in the present study, the presence of *Pseudomonas* was observed. According to various reports, *Pseudomonas* is involved in the breakdown of complex organic substances such as alkanes, alkenes, and polycyclic aromatic hydrocarbons into fatty acids like formate, lactate, acetate, ethanol, and CO_2_. Furthermore, the production of biosurfactants by the *Pseudomonas* genus can promote the breakdown of polycyclic aromatic hydrocarbons because these substances enable a decrease in surface and interfacial tensions between coal molecules to enhance solubility (Singh and Tripathi, 2013; Gründger et al., 2015; Colosimo et al., 2016; Mehboob et al., 2016; Davis and Gerlach, 2018). *Desulfotomaculum*, a methylotrophic sulfate-reducing bacteria found in our samples, has been previously reported in the formation water of the Jharia basin (Singh et al., 2012).

The results of the predictive functional analysis support the microbial diversity analysis, showing the presence of methanogenic functions in the reservoir. This indicates toward the potential for CBM enhancement in the studied wells. The highest methanogenic activity is observed in Well-E. The degradation of hydrocarbon compounds is an important step in the process of methanogenesis (Liu et al., 2022). A high abundance of aromatic degradation in Well-D, Well-E, and Well-A shows the potential of indigenous microbes to convert complex organic compounds into substrates used in the process of methane generation. The analysis reveals the existence of sulfate-reducing metabolism in the reservoir. Studies have also shown the coexistence of methanogens with sulfate-reducing bacteria in various environments (Sela-Adler et al., 2017). The presence of chitin and xylan degraders also represents the abundance of organic compounds degrading microbes, thus reflecting good potential of CBM enhancement in the wells.

**Figure 9:**
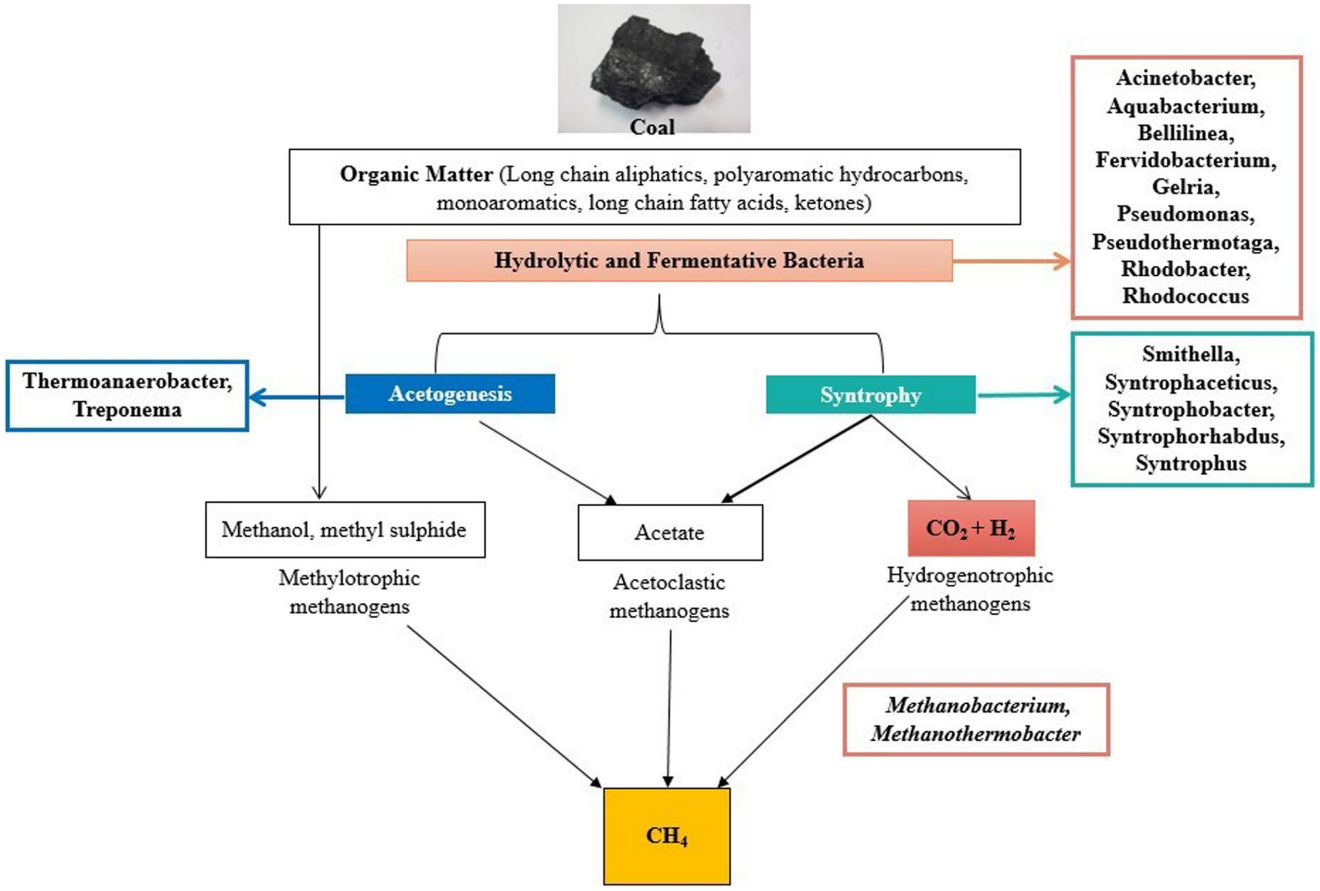
Pathway showing metabolism and microbes present in the formation water involved in coal degradation and generation of biogenic methane.

The predictive functional analysis is also supported by the flowchart diagram showing the pathway of degradation of coal into methane (Figure 9). The diagram showed various microbes present in the formation water that are known to be involved in various steps of coal degradation. The analysis revealed the abundance of hydrolytic, fermentative, and syntrophic microbes in the wells. These are the major key players in the process of methanogenesis (Nazaries et al., 2013). The analysis also revealed the presence of *Methanobacterium* and *Methanothermobacter* as the main methanogens in the wells; thus, hydrogenotrophic is the main route in the process of methane generation. *Acinetobacterium* is a well-known bacterial genus involved in the degradation of complex organic compounds (Zhang et al., 2021). The high abundance of Acinetobacter in the wells shows the capability of coal degradation in the wells. Genus *Fervidobacterium, Gelria*, and *Pseudomonas* are the hydrolytic and fermenting bacteria involved in methane generation (Harirchi et al., 2022). Genus *Rhodobacter* and *Rhodococcus* are involved in the degradation of various complex organic and inorganic compounds (Do et al., 2003; Margesin et al., 2005). Species of *Aquabacterium* are known to be involved in the biodegradation of oils; thus, they have the capability of degrading complex hydrocarbons (Wang et al., 2019). Genus *Thermoanaerobacter* and *Treponema* present in the wells are known to be involved in acetogenesis (Harirchi et al., 2022). Various syntrophic bacteria were found in the wells essential for methane generation (Figure 6).

Several biotic and abiotic factors play huge roles in the shaping of microbial communities (Wang et al., 2020; Rajeev et al., 2021). In the case of CBM wells, the physicochemical parameters of the formation water significantly impact the distribution and diversity of microbial communities in the sample (Davis and Gerlach, 2018). Previous studies have shown the impact of geochemical factors like salinity, temperature, and pH significantly impacting the diversity of methanogens in their natural environments (Hoehler et al., 2010; Wen et al., 2017). In the present study, the results of the canonical corresponding analysis identify the effect of geochemical factors on the microbial diversity of the reservoir. The analysis shows a relation between various classes found in the samples. A correlation analysis further corroborates this relation, indicating a significant correlation between Iron, Electrical Conductivity, and TDS with classes *Aminicenantia, Coprothermobacteria, Planctomycetacia, Thermodesulfovibriona*, and *Ignavibacteria*. Members of these classes play an important role in the initial step of methanogenesis. They are involved in hydrocarbon degradation, fermentative, and hydrogen-producing metabolism (Liu et al., 2012; Kadnikov et al., 2019; Pavan et al., 2019; Storesund et al., 2020). A study has shown the relation between high bulk conductivities and TDS with enhanced mineral weathering, interlinked with the activities of hydrocarbon-degrading microbes in aquifers contaminated with hydrocarbons (Atekwana et al., 2004). Another study has reported that Fe(III) can be used as an electron acceptor in microbial hydrocarbon degradation, zero-valent iron can donate electrons for improved methanogenesis, and conductive iron oxides may help electron transfers in methanogenic processes. These pieces of evidence show how the geochemical parameters can impact the metabolisms and distribution of hydrocarbon degraders in the ecosystem (Castro et al., 2022). These findings validate the results of the correlation analysis done in the present study.

To further emphasize the role and functions of the indigenous archaea and bacteria present in the CBM reservoir of the Raniganj block, indigenous microbes from the formation water were enriched in methanogen-specific nutrient media, and methane gas produced by the consortium via coal conversion was quantified. The results clearly indicate the real potential of the indigenous microbes present in the formation water for the bioconversion of coal into methane. They also confirm the capability of the nutrient media to support the growth of a selective methanogenic consortium that can utilize bituminous coal present in thermogenic CBM reservoirs as a carbon source and lead to *in situ* methane enhancement.

## 5. Conclusion

As per the authors’ knowledge, this is the first study that provides the microbial analysis of the thermogenic gas-producing CBM reservoir in the Raniganj block and its biogenic methane potential. The microbial diversity found in the formation water samples in this investigation comprised abundant syntrophic bacteria (e.g., *Coprothermobacter*), methylotrophic bacteria (e.g., *Methylobacterium, Methylobacillus*), fermentative bacteria (e.g., *Microbacterium, Pseudomonas, Acinetobacter*) and methanogenic archaea. Proteobacteria, Bacteroidetes, and Firmicutes were the three bacterial phyla that were consistent in all formation water samples, with Proteobacteria being the most prevalent of them. The archaeal domain at the genus level showed the presence of methanogenic archaea, including *Methanobacterium* and *Methanothermobacter*, which are two well-known hydrogenotrophic methanogens, indicating that the primary pathway of biogenic methane production in the Raniganj CBM block is hydrogenotrophic. This analysis lays the groundwork for greater comprehension of the functional pathways that predominate and the underlying geomicrobiological diversity, enabling us to examine how it affects methane generation on a lab scale. A deeper understanding of the processes at play could be beneficial in the development of strategies for enhanced methane production.

## Data availability statement

The datasets presented in this study can be found in online repositories. The names of the repository/repositories and accession number(s) can be found in the article/Supplementary material.

## Author contributions

ML conceptualized the complete project and designed the experiments. NSi and ML provided the resources to set up and perform the experiments. MC conducted all the experiments, recorded and analyzed the data, and wrote the original draft. NSa conceptualized the idea of metagenomic analysis. MC, NSa, and ML reviewed and edited the manuscript. SS and KS participated in sampling. AM, MI, and SK provided the geological data. All authors contributed to the article and approved the submitted version.

## Conflict of interest

AM, MI, and SK were employed by Essar Oil and Gas Exploration and Production Limited. SS and KS were employed by ONCG Energy Centre.

The authors declare that this study received financial support from Essar Oil and Gas Exploration and Production Limited (EOGEPL). The funder had the following involvement with the study: EOGEPL provided technical support in the manuscript, including well production history, and geographical and geological content of the studied CBM wells; Figures 1 and 2 in the manuscript were also provided by EOGEPL.

## Publisher’s note

All claims expressed in this article are solely those of the authors and do not necessarily represent those of their affiliated organizations, or those of the publisher, the editors and the reviewers. Any product that may be evaluated in this article, or claim that may be made by its manufacturer, is not guaranteed or endorsed by the publisher.
