# Supplementary material for: Culture-independent assessment of the indigenous microbial diversity of Raniganj coal bed methane block, Durgapur
Source: Front Microbiol. 2023 Sep 4;14:1233605. doi: 10.3389/fmicb.2023.1233605 (PMC10507629; doi:10.3389/fmicb.2023.1233605)
Supplement: Supplementary file 1 [file Image_1.pdf]

Culture-independent assessment of indigenous microbial diversity of Raniganj Coal Bed Methane block, Durgapur

Purpose of the study

A rarefaction curve was plotted between the species richness and the sequence sample size.

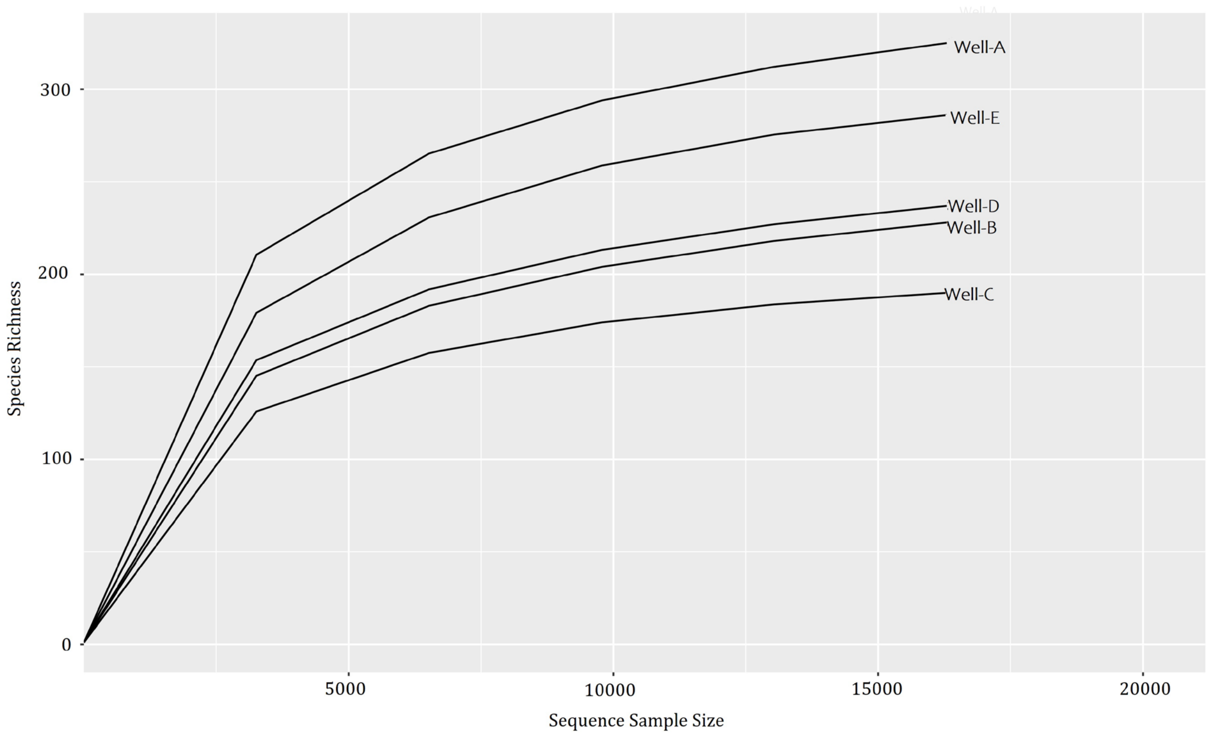

(Supplementary Fig. 1 Rarefaction curve depicting sequence richness and depth of the samples**)**

Results

The figure depicts that the curve reached a plateau after 15000 reads in the case of each well. This indicates that the depth of the sequencing was adequate, and the diversity of the microbial community was accurately estimated.
